# Studying Enhanced Recovery After Surgery (ERAS®) Core Items in Colorectal Surgery: A Causal Model with Latent Variables

**DOI:** 10.1007/s00268-020-05940-1

**Published:** 2021-02-11

**Authors:** Marco Gemma, Fulvia Pennoni, Marco Braga

**Affiliations:** 1 Anesthesia and Intensive Care, Fatebenefratelli Hospital, Piazza Principessa Clotilde, 3, 20121 Milan, Italy; 2 Department of Statistics and Quantitative Methods, University of Milano-Bicocca, Via degli Arcimboldi, 8, 20126 Milan, Italy; 3 Department of Surgery, University of Milano-Bicocca, San Gerardo Hospital, Via Pergolesi G. B., 33, 20900 Monza, Italy

## Abstract

**Background:**

Previous Enhanced Recovery After Surgery (ERAS®) studies have not always taken into account that ERAS interventions depend on baseline covariates and that several confounding variables affect the composite outcomes.

**Method:**

A causal latent variable model is proposed to analyze data obtained prospectively concerning 1261 patients undergoing elective colorectal surgery within the ERAS protocol. Primary outcomes (composite of any complication, surgical site infection, medical complications, early ready for discharge (TRD), early actual discharge) and secondary outcomes (composite of late bowel function recovery, IV fluid resumption, nasogastric tube replacement, postoperative nausea and vomiting, re-intervention, re-admission, death) are considered along with their multiple dimensions.

**Results:**

Concerning the primary outcomes, our results evidence three subpopulations of patients: one with probable good outcome, one with possibly prolonged TRD and discharge without complications, and the other one with probable complications and prolonged TRD and discharge. Epidural anesthesia, waiving surgical drainage, and early ambulation, IV fluid stop and urinary catheter removal act favorably, while preoperative hospital stay and blood transfusion act negatively. Concerning the secondary outcomes our results evidence two subpopulations of patients: one with high probability of good outcome and one with high probability of complications. Epidural anesthesia, waiving surgical drainage, early ambulation and IV fluid stop act favorably, while blood transfusion acts negatively also with respect to these secondary outcomes.

**Conclusion:**

The multivariate causal latent class two-parameter logistic model, a modern statistical method overcoming drawbacks of traditional models to estimate the average causal effects on the treated, allows us to disentangle subpopulations of patients and to evaluate ERAS interventions.

## Introduction

The ERAS (Enhanced Recovery After Surgery) is a multimodal perioperative care pathway intended to improve and shorten recovery after major surgery through the application of a bundle of interventions [1, 2]. However implementing all of the ERAS items is a hard work for any hospital and it is possible that some ERAS interventions exert a greater impact on outcome than others. Although the final goal is to realize a complete ERAS pathway, concentrating on some possible “core items” in the beginning could be prominently facilitating. Much interest is growing about the search for evidence on these core items [3].

Any study on this topic exploited the incomplete compliance with ERAS items, which variably accompany ERAS databases and provides the necessary variability for addressing the question of the benefit of a single item or of ERAS as a whole. Nevertheless, previous analysis did not adequately consider that non-compliance is scarcely ever independent from other important variables. This raises at least three major methodological issues that have been poorly addressed in previous studies. First, ERAS outcomes and ERAS interventions themselves are affected by several confounding variables. For example, the American Society of Anesthesia score (ASA) status can affect both the outcome and the early postoperative mobilization. Second, ERAS items are themselves inter-related. For example, conservative intraoperative fluid administration is conceivably more applicable in patients who did not receive preoperative bowel preparation. Third, when dealing with ERAS, the outcome measures are composite. For example, the principal outcome measures in ERAS studies, postoperative length of hospital stay and complications, cannot be considered separately since they measure a similar trait.

We retrospectively studied a prospectively collected data of patients undergoing elective colorectal surgery between 2014 and 2018 with an ERAS protocol in 20 Italian hospitals affiliated with the PeriOperative Italian Society (POIS). The aim of our study is to analyze the effects of a number of ERAS items with a statistical model that considers the aforementioned methodological issues, namely a multivariate Latent Class Two-Parameter Logistic (LC-2PL) model [4, 5] formulated within a potential outcome framework [6]. We estimate the Average Causal Effects on the Treated (ATET) adequately weighting each patient through the Inverse-Probability-of-Treatment (IPT, [7]). As recently remarked by [8] propensity score methods allow us to block the association between observed confounding variables and treatments, thus permitting to reduce bias due to pre-treatment imbalances in observational studies. Innovatively we propose to estimate patient’s weights according to the pre-treatment covariates and sequential blocks of treatments. We summarize the responses by means of a multivariate latent variable model suitable to classify patients and to assess the effects of the ERAS interventions on primary and secondary outcomes.

## Materials and methods

### Study design

Twenty Italian hospitals affiliated with POIS collaborated in collecting data. All centers treated their patients within an ERAS pathway, which was defined with active contribution from the ERAS Society. Before the start of the study, all hospitals had been involved in a pathway implementation program led by POIS consisting in education and audit meetings every six months for a two-year period.

All data were collected prospectively through a standardized electronic spreadsheet, which was used to record 90 variables per patient [9]. Every three months, the center-specific spreadsheets containing data collected in that time period were merged into a web-based password-protected database managed by POIS. Data collected included demographics, patient comorbidities, preoperative and intraoperative variables, adherence to ERAS items, early recovery variables, and short-term postoperative outcomes.

**Fig. 1:**
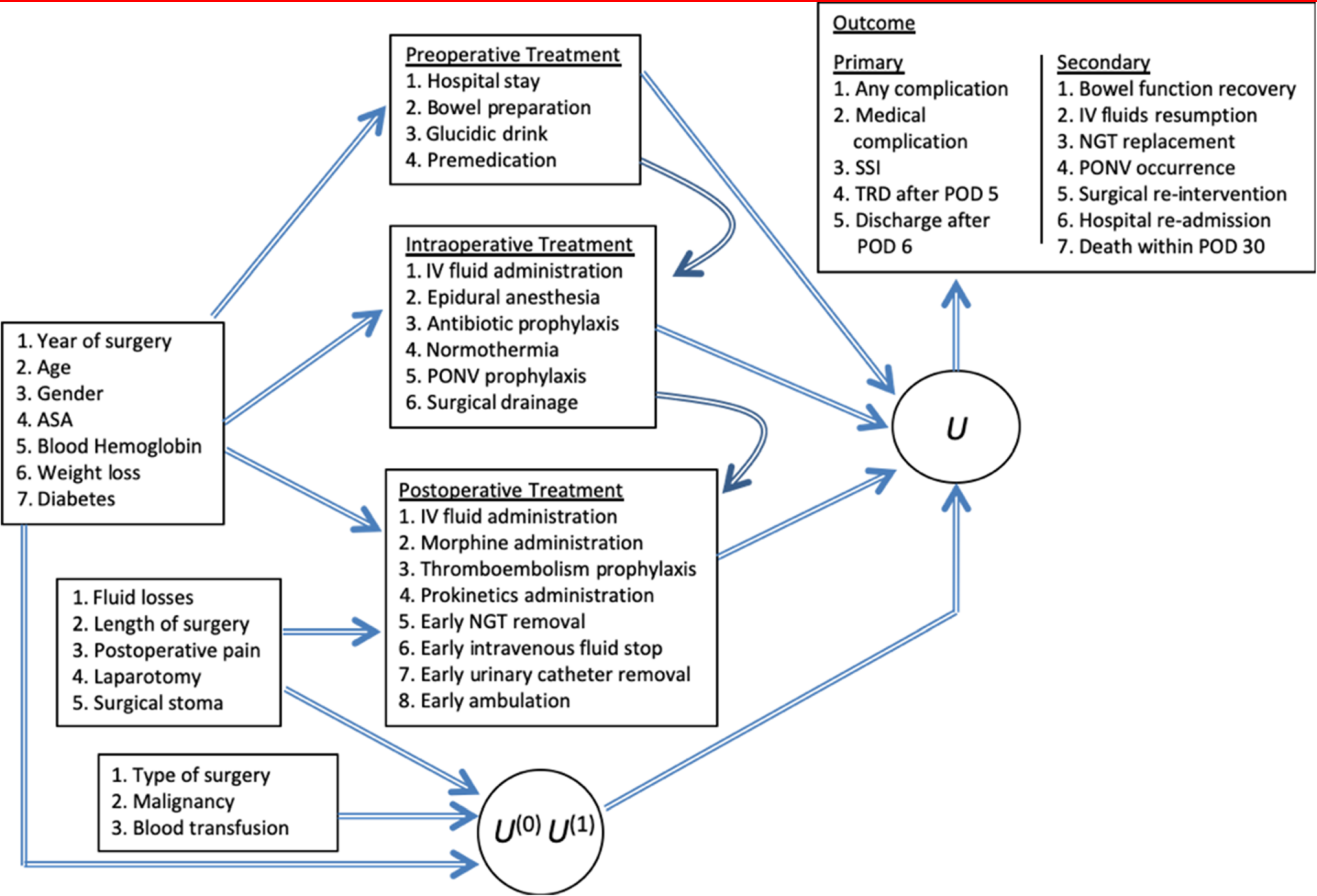
Conceptual framework of the proposed multivariate causal latent variable model. IV: intravenous; NGT: nasogastric tube; PONV: postoperative nausea and vomiting; ASA: American Society of Anesthesia score; SSI: surgical site infection; TRD: time ready for discharge. *U*: latent variable; *U*^(0)^: latent potential outcome referred to the non-treated patient; *U*^(1)^: latent potential outcome referred to the treated patient

Figure 1 shows the conceptual framework used to define sequentially the treatments, the confounders, the potential outcomes, the latent variables and the observed outcomes. We study 18 items out of the POIS database. For most of these treatments ERAS recommendations are available. Since our purpose is to account for the aforementioned inter-relationship between these treatments, we grouped them according to the phase of the patient’s pathway in which they are applied. Hence, treatments are classified in three consecutive units, namely preoperative, intraoperative, and postoperative, and each unit affects the ones to follow. Four treatments are considered as preoperative, namely preoperative hospital stay (number of days), no bowel preparation, glucidic drink administration, and premedication. Six are considered as intraoperative, namely IV fluid administration (ml/kg/h), epidural anesthesia, antibiotic prophylaxis, maintenance of normothermia, nausea and vomiting (PONV) prophylaxis, and no surgical drainage. Eight treatments are considered as postoperative, namely intravenous fluid administration (ml/kg during POD 1), morphine administration (dichotomous no/yes variable without differentiating between different administration modes—PCA, elastomer, fixed-dose—or different dosing), thromboembolism prophylaxis, prokinetic administration, naso-gastric tube (NGT) removal within POD 0, intravenous fluid stop within POD 2, urinary catheter removal within POD 1, and ambulation within POD 1. The cut-off POD choice for the last four variables has been decided according to the clinical experience.

There are 15 observed confounders. Seven of these potentially affect compliance with all of the treatments: year of surgery, age, gender, ASA classification of general health status (ASA1–2 vs ASA > 2), baseline blood hemoglobin (mg/dl), preoperative body weight loss, and preoperative diagnosis of diabetes mellitus. Five confounders potentially affect compliance only with postoperative treatments: intraoperative fluid losses (ml/kg), length of surgery (min), maximum postoperative pain on POD 1–4 [measured on the Numerical Rating Scale (NRS)], laparotomy (vs laparoscopy), and surgical stoma (coded as binary variables). We also consider the following external variables that directly affect the outcomes: type of surgery (colonic or rectal), malignancy of the underlying disease, blood derivatives transfusion.

The main advantage of the proposed causal latent class model is that we jointly account for several outcomes that are distinguished as primary and secondary according to their relative clinical importance in the ERAS framework. The primary outcomes are the following: occurrence of any complication, occurrence of surgical site infection (SSI), occurrence of medical complications unrelated to the surgical site, such as cardiovascular, pulmonary, thromboembolic, or urinary complications, ready for discharge after POD 5, and actual discharge from hospital after POD 6 [10]. Within our proposal, as illustrated in the next section, we are able to consider the outcomes jointly and to account for the fact that they mainly concern two dimensions: the first is made by complications and the second one is made by Time Ready for Discharge (TRD) and actual discharge.

The secondary outcomes that we account jointly are the following: bowel function recovery after POD 1, need for IV fluid resumption after suspension, need for nasogastric tube (NGT) replacement after removal, occurrence of postoperative nausea and vomiting (PONV), surgical re-intervention, hospital re-admission, death within POD 30. In the multivariate model presented below we assume they represent two distinct dimensions: the first made by bowel function, need for IV fluid resumption, need for nasogastric tube, nausea and vomiting and the second made by surgical re-intervention, hospital re-admission and death.

### Statistical model

We highlight a proposal for the estimation of multiple treatments within observational studies, since we are interested to disentangle the effects of the ERAS items on primary and secondary outcomes. The treatment is confounded with the patient’s characteristics and to address this problem we follow the potential outcome approach to causal inference as proposed by [11, 12] and we extend the IPT weighed estimator [13, 14] to a multivariate LC-2PL model [15]. The estimation of the probability to be treated is made sequentially along pre-treatment covariates and blocks of treatments (see Fig. 1).

Differently from previous proposals we suppose the counterfactual outcomes as latent variables denoted as $U_{i}^{\left( 0 \right)}$ and $U_{i}^{\left( 1 \right)}$ indicating for each patient $i,i = 1, \ldots ,n$*,* the variable under the non-treated and treaded status, respectively. An underlying latent variable $U_{i}$ is assumed to depend on both $U_{i}^{\left( 0 \right)}$ and $U_{i}^{\left( 1 \right)}$ as well as on the treatments (see Fig. 1). We assume local independence [5, 16] meaning that the observed outcomes are conditionally independent given the potential latent variables and the treatments administered sequentially.

In what follows, first we describe the estimation of the probability of treatment exposure given the pre-treatment covariates and each block of ERAS items (according to the arrows depicted in Fig. 1). This estimation is made by a sequence of linear or logistic regressions. Next, we calculate weights for each patient as the inverse of the probability of the observed treatment sequence. Third, we use stabilized weights to estimate a weighted causal LC-2PL model on the primary and secondary outcomes. The estimation is carried out through the maximization of a weighted log-likelihood employing the Expectation–Maximization algorithm (EM [17]). We rely on the Bayesian Information Criterion [BIC 18] through which we choose the suitable number of latent components. Fourth, we estimate the selected model by adding the covariates through a convenient parameterization considering the first latent class as reference since it identifies the subpopulation of patients recovered as expected.

The latent class model [4, 5] first proposed by [19] to classify units within a probabilistic approach is formulated as a finite mixture model [20]. Following some recent proposals in the literature, we formulate a multivariate LC-2PL model [21] to infer causal effects within observational studies [15, 22, 23]. We introduce a novel use of this model by attempting to estimate the effects of various sequential interventions on patients entered in the ERAS project. We model the marginal distribution of the counterfactual variables [6, 24, 25] and as a result the estimated regression coefficient encode the magnitude of the ATET [8]. In this way we mimic an artificial random assignment scenario essential to account for differences among patients. As pointed out by [7] the use of the Inverse-Probability-of-Treatment Weighting [IPTW 26] allow us to disentangle the association between the observed confounding variables and treatments thus permitting to reduce bias due to confounding.

### A causal latent variable model

The potential outcomes of the patient are usually referred to as $Y_{i}^{\left( 1 \right)}$ if the patient is exposed to treatment $Z_{i}$ and as $Y_{i}^{\left( 0 \right)}$ if the patient is not exposed with $i$, $i = 1, \ldots ,n$. The treatment effect is given by the difference $Y_{i}^{\left( 1 \right)} - Y_{i}^{\left( 0 \right)}$ and the expected value of this difference over the entire population of treated patients is defined as the ATET. We instead postulate the existence of the underlying latent potential variables denoted as $U_{i}^{\left( z \right)}$ and we define the ATET as

$$ {\text{ATET}} = {\text{E}}\left( {U_{i}^{\left( 1 \right)} - U_{i}^{\left( 0 \right)} |Z_{i} = 1{ }} \right), $$

for $i$, $i = 1, \ldots ,n$, and of the latent variable $U_{i}$ depending on the treatment through the latent potential variables as follows

$$ U_{i} = \left( {1 - Z_{i} } \right)U_{i}^{\left( 0 \right)} + Z_{i} U_{i}^{\left( 1 \right)} . $$

For this latent variable we assume a discrete distribution left unspecified with a finite number of support points ranging from 1 to *k*.

Let $Y_{ir}$ be the observed binary response referred to outcome $r, r = 1, \ldots ,p$ for each patient $i,i = 1, \ldots ,n,$ randomly drawn from the population. Following [27, 28] we extend the proposal of [15] to estimate patient specific weights. We formulate the following assumptions: *i*) conditional exchangeability meaning that the latent potential outcomes are independent on the treatment given the covariates, *ii*) positivity (ignorable treatment assignment) meaning that there is a positive probability for every patient of receiving any type of treatment, *iii*) consistency implying that the latent potential variables are well-defined and as a result any observed outcome is the potential variable corresponding to the observed treatment sequence.

Let ${\varvec{x}}_{i}$ denotes the covariates for patient $i$ observed prior to the treatment assignment, the weight for this patient corresponds to the inverse of the conditional probability of receiving the treatment. In the case of a binary treatment we use the following logit model to estimate this probability

$$ p_{i} {\text{ = log}}\frac{{P\left( {Z_{i} = 1{|}{\varvec{x}}_{i} } \right)}}{{P\left( {Z_{i} = 0{|}{\varvec{x}}_{i} } \right)}} = \alpha + {\varvec{x}}_{i}^{^{\prime}} {\varvec{\gamma}}, $$

where $$\alpha$$ and $$\varvec{\gamma ^{\prime}}$$ denotes the intercept and the vector of regression coefficients respectively. The weights are estimated sequentially according to the blocks of the ERAS items illustrated in Fig. 1. The arrows reported in this figure pointing from the risk factors into the other blocks indicate that the treatment is confounded and causally endogenous. The overall weight of patient $$i $$ is determined as the sum of the product of the estimated inverse-probabilities of the treatments in each block as follows

$$ \hat{w}_{i} = \mathop \sum \limits_{v = 1}^{V} \frac{1}{{\mathop \prod \nolimits_{j = 1}^{J} p_{ij} }}, $$

where $v = 1, \ldots ,V$ denotes the block and $j, j = 1, \ldots ,J$ denotes the treatment in each block. The weights are stabilized by trimming them up to certain level to avoid high variability [23].

The dependence of the potential latent variables is modelled through the following multinomial logit model

$$ {\text{log}}\frac{{p\left( {U_{i}^{\left( z \right)} = u} \right)}}{{p\left( {U_{i}^{\left( z \right)} = u - 1} \right)}} = \beta_{0u} + {\varvec{d}}\left( z \right)\user2{^{\prime} {\varvec{\beta}} }_{1u} $$

where $u = 2, \ldots ,k,$ $\beta_{0u}$ is the intercept specific of each latent class, ${\varvec{d}}\left( z \right)$ is a vector with elements equal to 1 for treated patients and ${\varvec{\beta}}_{1u}$ is the vector of parameters that define the ATET in the distribution of the latent variables.

Another set of parameters is referred to the conditional distribution of the observed outcomes and it is defined as

$$ p\left( {Y_{ir} = 1{|}U_{i}^{\left( z \right)} = u} \right) = \frac{1}{{1 + {\text{exp}}\left[ { - \eta_{r} \left( {\xi_{i} - \delta_{r} } \right)} \right]}} , $$

where $u = 1, \ldots ,k,$ $\eta_{r}$ is a parameter measuring the discriminating power of the outcome $r,r = 1, \ldots ,p$, $\delta_{r}$ is another parameter measuring the difficulty of the outcome, and $\xi_{i}$ indicates the point on the latent continuum where patient $i,i = 1, \ldots ,n$ is located. This is a 2PL model specification for the dichotomously scored outcomes.

The model is estimated through a weighted log-likelihood function by considering a sample of $n$ independent patients for which we observe the multivariate binary outcomes. The target log-likelihood function is

$$ l\left( {\varvec{\theta}} \right) = \mathop \sum \limits_{i = 1}^{n} \hat{w}_{i} l_{i} \left( {\varvec{\theta}} \right), $$

where $\varvec{\theta}$ denotes the overall vector of free parameters arranged in a suitable way. This weighted log-likelihood is maximized through the EM algorithm [17]. The latter estimates the model parameters considering as missing data the vector of latent variables. Then E-step of the algorithm computes the conditional expected value of the complete-data log-likelihood given the observed data and the current value of the parameters. The M-step updates the parameters maximizing the expected value of the quantity computed at the E-step. The two steps are alternated repeatedly until a convergence criterion is reached.

In order to choose the best number of latent classes and to discover meaningful groups of patients in the population we apply a model selection strategy. The resulting subpopulations should be internally cohesive and well separated from one another. We rely on the BIC index [18] that is a measure of the relative goodness of fit of the model able to account simultaneously for its accuracy and complexity. It is defined as

$$ {\text{BIC}} = - 2l( {\hat{\varvec{\theta }}}) + {\text{log}}\left( n \right) \# {\text{par}}, $$

where $l ({\hat{\varvec{\theta }}})$ denotes the maximum of the weighted log-likelihood of the model with *k* latent classes, #${\text{par}}$ denotes the number of free parameters and *n* is the sample size. The model is estimated for an increasing number of latent classes and the best model is selected as that before the BIC starts to increase. Once the suitable number of latent classes is selected every patient is allocated to a latent class according the highest posterior probability. Standard errors for the estimated parameters are obtained as the square root of the diagonal elements of the inverse of the observed or expected information matrix computed through numerical methods.

## Results

The available observations are related to 1261 patients operated between 2014 and 2018. Table 1 shows some descriptive statistics. The model is estimated by using the open source software R [29] through the package multiLCIRT [30]. As far as we know there are no other software able to account for the multidimensionality of the responses and latent variables with a discrete distribution. For replicability purposes the code to estimate the proposed model is available from the repository at the following link https://github.com/penful/Eras. The complete results are available from the authors upon request.

**Table 1:** Frequency distribution of the available data

*Characteristics*	
Colon (vs rectal) surgery	962 (76.29)
Malignant lesion	1058 (83.90)
Blood transfusion	116 (9.20)
*Preoperative treatment*	
Preoperative hospital stay (days)	1.56 ± 3.56
Bowel preparation	160 (12.69)
Glucidic drink	887 (70.34)
Premedication	522 (41.40)
*Intraoperative treatment*	
IV fluids (ml/kg/h)	8.95 ± 4.43
Epidural anesthesia	468 (37.11)
Antibiotic prophylaxis	1243 (98.57)
Maintenance of normothermia	1243 (98.57)
PONV prophylaxis	940 (74.54)
Surgical drainage	862 (68.36)
*Postoperative treatment*	
IV fluids on POD 1 (ml/kg)	21.57 ± 11.24
Morphine	458 (36.32)
Thrombosis prophylaxis	1250 (99.13)
Prokinetics	368 (29.18)
Removal of NGT (POD)	0.16 ± 0.73
Stop of IV fluids (POD)	2.52 ± 2.52
UC removal (POD)	1.74 ± 1.96
Ambulation within POD 1	1043 (82.71)
*Covariates*	
Age (yrs)	67.25 ± 11.78
Male gender	676 (53.61)
ASA > 2	441 (34.97)
Baseline serum hb (mg/dl)	12.86 ± 1.98
Preoperative weight loss	84 (6.66)
Diabetes mellitus	162 (12.85)
Intraoperative fluid loss (ml/kg)	4.81 ± 2.60
Length of surgery (min)	203.26 ± 75.63
Max pain on POD 1–4 (NRS)	3.55 ± 2.02
Laparotomy	251 (19.90)
Surgical stoma	192 (15.23)
*Primary outcomes*	
Any complication	259 (20.54)
Medical complication	67 (5.31)
SSI	91 (7.22)
TRD after POD 5	6.26 (4.75)
Discharge after POD 6	6.6 (4.64)
*Secondary outcomes*	
Bowel function recovery (POD)	1.9 (1.11)
IV fluids resumption	158 (12.53)
NGT replacement	96 (7.61)
PONV occurrence	133 (10.55)
Surgical re-intervention	59 (4.68)
Hospital re-admission	23 (1.82)
Death within POD 30	7 (0.56)

ASA: American Society of Anesthesia status score; PONV: postoperative nausea and vomiting; POD: postoperative day; NGT: naso-gastric tube; SSI: surgical site infection; hb: serum hemoglobin; IV: intravenous; UC: urinary catheter; TRD: time ready to discharge. Categorical variables are reported as number (percentage), continuous variables as mean ± standard deviation. The year of the surgery is omitted from the table

### Results for the primary outcomes

The multivariate causal LC-2PL model is estimated for a number of latent classes ranging from 1 to 5. According to the lowest value of the BIC index we select the model with three latent classes. Table 2 reports on the estimated conditional probabilities of the primary outcomes. The first class, which encompasses 47% of the patients, presents low probability of complications and of late discharge. The second class, representing 35% of the patients, exhibits a high probability of late TRD and actual discharge although complications are improbable. Patients in the third class, accounting for the remaining 18% of patients, present high probability of complication and late TRD and actual discharge. Since these subpopulations are ordered according to the outcome occurrence we define the first LC as that representing the subpopulation of the best performing patients, the second that of intermediate patients and the third that of worst performing patients.

**Table 2:** Estimated probability of each latent class and estimated conditional probabilities of the multivariate causal latent class two-parameter logistic model for the primary outcomes

	Latent class		
	1	2	3
Estimated probability of each Latent Class	0.47	0.35	0.18
Estimated conditional probability for primary outcomes			
Any complication	0.07	0.08	0.82
SSI	0.01	0.01	0.35
Medical complication	0.02	0.02	0.28
TRD after POD 5	0.00	0.94	0.95
Discharge after POD 6	0.00	0.65	0.87

SSI: surgical site infection; TRD: time ready for discharge; POD: postoperative day. Any complications, SSI and medical complication measure the first dimension, TRD after POD 5 and Discharge after POD 6 measure the second dimension

By looking at the estimated ATETs defined with respect to the first LC chosen as reference due to the fact that it collects patients with the best outcomes, we evaluate the efficacy of each intervention. Table 3 reports on the estimated ATETs of being in the 1st rather than in the 2nd LC. The estimated regression coefficients in the upper part of the table indicate that no bowel preparation, colon surgery, ambulation within POD 1, IV fluid stop within POD 2, urinary catheter removal within POD 1, epidural anesthesia, and not inserting any surgical drainage significantly favor being in the class of best performing patients (1st LC) rather than in the group of less (intermediate) performing patients (2nd LC).

**Table 3:** Estimated effects of the causal latent class two-parameter logistic model for being in the 1st latent class rather than in the 2nd latent class for the primary outcomes. Estimated standard errors and asymptotic confidence interval at confidence level of 0.95

1st versus 2nd Latent Class	Estimated coefficient	S.E.	CI	
Intercept	1.49	1.08	(− 0.63, 3.61)	
No bowel preparation	− 0.85*	0.51	(− 1.21, −0.43)	Favor being in the 1st LC
Colon surgery	− 0.82***	0.20	(− 1.85, 0.15)	
Ambulation within POD 1	− 0.76***	0.21	(− 1.17, −0.35)	
IV fluid stop within POD 2	− 0.75***	0.17	(− 1.08, −0.42)	
UC removal within POD 1	− 0.62***	0.19	(− 0.99, −0.25)	
Epidural anesthesia	− 0.39**	0.18	(− 0.74, −0.04)	
No surgical drainage	− 0.35*	0.18	(− 0.70, 0.00)	
Prokinetics	− 0.15	0.16	(− 0.46, 0.16)	
NGT removed on POD 0	− 0.15	0.27	(− 0.68, 0.38)	
Morphine	− 0.05	0.16	(− 0.36, 0.26)	
Preoperative glucidic drink	− 0.04	0.16	(− 0.35, 0.27)	
Intravenous fluids	− 0.02	0.02	(− 0.06, 0.02)	
IV fluids during POD 1	0.01	0.01	(− 0.01, 0.03)	Favor being in the 2nd LC
Preoperative hospital stay	0.05**	0.02	(0.01, 0.09)	
PONV prophylaxis	0.08	0.19	(− 0.29, 0.45)	
Premedication	0.40	0.24	(− 0.07, 0.87)	
Malignant lesion	0.45**	0.20	(0.06, 0.84)	
Thromboembolism prophylaxis	0.69	0.90	(− 1.07, 2.45)	
Blood transfusion	1.16**	0.32	(0.53, 1.79)	

LC: latent class; IV: intravenous; UC: urinary catheter; NGT: nasogastric tube; POD: postoperative day. Significance levels for the test based on the estimated standard errors that each parameter is equal to zero: *significant at 1%; **significant at 5%; ***significant at 10%

The estimated regression coefficients in the bottom part of the table indicate that preoperative hospital stay, malignant lesion, and blood transfusion significantly favor being in the group of intermediate performing patient (2nd LC) with respect to best performing (1st LC).

Table 4 reports on the estimated ATETs of being in the 2nd rather than in the 3rd LC. The estimated regression coefficients in the upper part of the table indicate that ambulation within POD 1, epidural anesthesia, IV fluid stop within POD 2, urinary catheter removal within POD 1, and not inserting any surgical drainage favor being in the class of intermediate performing patients (2nd LC) rather than in the group of worst performing patients (3rd LC).

**Table 4:** Estimated effects of the causal latent class two-parameter logistic model for being in the 2nd rather than in the 3rd latent class for the primary outcomes. Estimated standard errors and asymptotic confidence interval at confidence level of 0.95

2st versus 3rd Latent Class	Estimated coefficient	S.E.	CI	
Intercept	2.08*	1.14	(− 0.15, 4.31)	
Ambulation within POD 1	− 1.51***	0.28	(− 2.06, − 0.96)	Favor being in the 2nd LC
Thromboembolism prophylaxis	− 1.12	0.83	(− 2.75, 0.51)	
Epidural anesthesia	− 0.90***	0.27	(− 1.43, − 0.37)	
IV fluid stop within POD 2	− 0.81***	0.24	(− 1.28, − 0.34)	
UC removal within POD 1	− 0.68**	0.26	(− 1.19, −0.17)	
No surgical drainage	− 0.67**	0.26	(− 1.18, − 0.16)	
No bowel preparation	− 0.48	0.72	(− 1.89, 0.93)	
Colon surgery	− 0.36	0.28	(− 0.91, 0.19)	
Prokinetics	− 0.31	0.25	(− 0.80, 0.18)	
NGT removed on POD 0	− 0.08	0.36	(− 0.79, 0.63)	
Intraoperative fluids	− 0.04	0.03	(− 0.10, 0.02)	
IV fluids during POD 1	0.02**	0.01	(0.00, 0.04)	Favor being in the 3rd LC
Preoperative hospital stay	0.05**	0.02	(0.01, 0.09)	
Preoperative glucidic drink	0.20	0.22	(− 0.23, 0.63)	
Morphine	0.24	0.21	(− 0.17, 0.65)	
Malignant lesion	0.38	0.28	(− 0.17, 0.93)	
PONV prophylaxis	0.54**	0.28	(− 0.01, 1.09)	
Premedication	0.97***	0.33	(0.32, 1.62)	
Blood transfusion	1.90***	0.35	(1.21, 2.59)	

LC: latent class; IV: intravenous; UC: urinary catheter; NGT: nasogastric tube; POD: postoperative day. Significance levels for the test according to the estimated standard errors that each parameter is equal to zero: *significant at 1%; **significant at 5%; ***significant at 10%

The estimated regression coefficients in the bottom part of the table indicate that postoperative fluids, preoperative hospital stay, PONV prophylaxis, premedication, and blood transfusion significantly favor being in the group of worst performing patients (3rd LC) rather than stay in the intermediate group of patients (2nd LC).

### Results for the secondary outcomes

The multivariate causal LC-2PL model for the secondary outcomes is estimated as the previous model for a number of latent classes ranging from 1 to 5. According to the lowest value of the BIC index we select the model with two latent classes. The estimates of the model parameters for the secondary outcomes are reported in Tables 5 and 6. According to the results showed in Table 5 we notice that both latent classes have a similar probability of bowel function recovery after POD 1 (0.51 and 0.56 respectively) and a null or very low probability of death before POD 30 (0.00 and 0.01 respectively). The first class (78% of patients) exhibits low probability for all the other secondary outcomes. In the second latent class IV fluid resumption, NGT replacement, PONV occurrence and surgical re-intervention are sensibly more probable, while hospital re-admission is only slightly more probable.

**Table 5:** Estimated probability of each latent class and estimated conditional probabilities of the multivariate causal latent class two-parameter logistic model for the secondary outcomes

		Latent class	
		1	2
Estimated probability of each Latent Class		0.78	0.22
Estimated conditional probability for secondary outcomes	Bowel function recovery after POD 1	0.51	0.56
	IV fluid resumption	0.03	0.57
	NGT replacement	0.00	0.34
	PONV occurrence	0.08	0.41
	Surgical re-intervention	0.01	0.16
	Hospital re-admission	0.00	0.07
	Death within POD 30	0.00	0.01

POD: postoperative day; IV: intravenous; NGT: nasogastric tube; PONV: postoperative nausea and vomiting measure the first dimension and the remaining outcomes measure the second dimension

**Table 6:** Estimated effects of the causal latent class two-parameter logistic model for being in the 1st latent class rather than in the 2nd latent class for the secondary outcomes. Estimated standard errors and asymptotic confidence interval at confidence level of 0.95

1st vs 2nd LC	Estimated coefficient	S.E.	CI	
Intercept	1.24	1.37	(− 1.45, 3.93)	
Thromboembolism prophylaxis	− 2.56***	0.80	(− 4.13, − 0.99)	Favor being in the 1st LC
Ambulation within POD 1	− 1.48***	0.27	(− 2.01, − 0.95)	
IV fluid stop within POD 2	− 1.45***	0.25	(− 1.94, − 0.96)	
NGT removed on POD 0	− 1.21***	0.36	(− 1.92, − 0.50)	
No bowel preparation	− 0.77	0.92	(− 2.57, 1.03)	
No surgical drainage	− 0.48*	0.27	(− 1.01, 0.05)	
Epidural anesthesia	− 0.47*	0.27	(− 1.00, 0.06)	
Colon surgery	− 0.23	0.28	(− 0.78, 0.32)	
PONV prophylaxis	− 0.13	0.28	(− 0.68, 0.42)	
UC removal within POD 1	− 0.04	0.26	(− 0.55, 0.47)	
Intraoperative fluids	− 0.02	0.03	(− 0.08, 0.04)	
Morphine	− 0.02	0.23	(− 0.47, 0.43)	
IV fluids during POD 1	− 0.01	0.01	(− 0.03, 0.01)	
Preoperative hospital stay	0.00	0.03	(− 0.06, 0.06)	Favor being in the 2nd LC
Malignant lesion	0.04	0.30	(− 0.55, 0.63)	
Premedication	0.46	0.38	(− 0.28, 1.20)	
Blood transfusion	0.87**	0.33	(0.22, 1.52)	
Preoperative glucidic drink	0.97**	0.28	(0.42, 1.52)	
Prokinetics	1.22**	0.20	(0.75, 1.69)	

LC: latent class; IV: intravenous; UC: urinary catheter; NGT: naso-gastric tube; POD: postoperative day. Significance levels for the test based on the estimated standard errors that each parameter is equal to zero: *significant at 1%; **significant at 5%; ***significant at 10%

Table 6 reports on the estimated ATETs of being in the 1st rather than in the 2nd LC. The estimated regression coefficients in the upper part of the table indicate that thromboembolism prophylaxis, ambulation within POD 1, IV fluid stop within POD 2, NGT removal on POD 0, not inserting any surgical drainage, and epidural anesthesia favor being in the class of best performing patients (1st LC) rather than in the group of the worst performing patients (2nd LC).

The estimated ATETs in the bottom part of the table indicate that blood transfusion, preoperative glucidic drink, and prokinetics significantly favor being in the group of the worst performing patient (2nd LC) with respect to best performing (1st LC).

## Discussion

With respect to our primary outcomes, the model points out three classes of patients. The first one has a high probability of good outcome and represents the majority of our patients. The second class exhibits possibly prolonged TRD and discharge notwithstanding the absence of complications. The third class presents high probability of both complications and prolonged TRD and discharge. Five variables positively affect the outcomes (ambulation within POD 1, IV fluid stop within POD 2, urinary catheter removal within POD 1, epidural anesthesia, and not inserting any surgical drainage). Two variables negatively affect the outcomes (preoperative hospital stay and blood transfusion).

With respect to our secondary outcomes, three of them could not contribute to discrimination between classes of patients, since their probability was uniformly either low (hospital re-admission and death within POD 30) or high (bowel function recovery after POD 1). The model points out two classes of patients: a first class with high probability of good outcome (including the majority of our patients), and a second one with high probability of IV fluid resumption, NGT replacement, PONV occurrence, and surgical re-intervention. Six variables positively affect the secondary outcomes and four of them do the same for the primary outcomes (ambulation within POD 1, IV fluid stop within POD 2, epidural anesthesia, and not inserting any surgical drainage). Four variables negatively affect the secondary outcomes and one of them does the same for the primary outcomes (blood transfusion).

Our results contribute to the ongoing debate about the existence of ERAS “core-items”. In fact, although it is recognized that the complete ERAS protocol is the best way to improve postoperative outcome, the number and relative combination of the ERAS items implemented in previous works varied across studies without substantial differences in postoperative short-term outcomes [31, 32]. Indeed, several studies imply that some ERAS elements may be more significant than others in affecting outcome [33–36] and that simplified protocols could yield comparable results [23, 24]. A systematic review suggests that the number of implemented ERAS items does not affect outcome and that applying only some core-items is sufficient to obtain the benefits of the ERAS program [37]. In contrast, a large cohort of patients from the ERAS Society Registry suggested that the improvement in clinical outcome provided by an ERAS program was directly correlated with the number of implemented items and the degree of compliance to the protocol [38, 39].

In addressing these issues no previous study adequately considered the dependency of multivariate outcomes from confounding variables and non-compliance to ERAS items, the inter-relation between ERAS items themselves, and the composite nature of the primary and secondary outcomes at stake.

The estimation of treatment effects in observational studies when the treatment assignment depends on the sequence of previous assignments and on time-varying confounders is still a matter of debate. The main advantage over the standard simple multinomial logit model is that our counterfactual framework assesses causal associations, corrects for pre-treatment confounders and for multiple treatment conditions in order to reduce the bias due to confounding. Another advantage is that it is a multivariate model-based clustering method and allows us to properly account for suitable groups able to disentangle the heterogeneous population of patients. Moreover, it yields a classification that uses the maximum a-posteriori estimates of the model parameters.

The results of the application provide evidence that waiving bowel preparation increases the probability of good outcome. Recent ERAS guidelines on colonic surgery and a large meta-analysis of more than 21,000 patients agree that mechanical bowel preparation is not associated to any reduction in postoperative complications, mortality, and length of hospital stay when compared with no preparation [40, 41]. Actually, waiving preoperative mechanical bowel preparation reduces the risk of preoperative dehydration and the possibly associated electrolyte disorders. This favors a reduction in fluid administration and the reaching of zero fluid balance. Moreover, a possible increment in Gram-negative bacterial components of the intestinal flora is associated with bowel preparation [42].

Recent studies demonstrate how a balanced intraoperative goal directed therapy reduces mortality, overall morbidity, and the time to first flatus and to oral feeding [43]. The ERAS guidelines clearly state that postoperative IV fluid administration is not necessary if oral intake is possible and that early oral feeding is safe and well tolerated by the majority of patients after colorectal surgery [44]. Early stop of IV fluid infusion and urinary catheter removal, together with good pain control and no surgical drainage, foster patients’ mobilization after surgery. A reduction of postoperative pulmonary and thromboembolic complications and a regain of preoperative functional capacity and muscular strength are strictly related to early postoperative mobilization [45].

The results suggest that epidural analgesia exerts a favorable effect on outcome and therefore they confirm the findings of other studies according to which epidural analgesia after laparotomy provides optimal pain control and reduces the inflammatory stress response. This reduces the incidence of pulmonary and cardiovascular complications, in particular in frail patient [46, 47]. Nevertheless, the role of thoracic epidural analgesia after laparoscopic procedures is controversial and the importance of multimodal analgesic sparing-opioids strategies is widely accepted in less invasive surgery. Studies addressing purely laparoscopic colonic surgery suggest caution towards epidural analgesia [48]. It should be noted that, although laparoscopy is widely recognized as a predictor of faster recovery, this effect was not apparent in our results.

The negative effect of a prolonged preoperative hospital stay, which we observed, deserves attention. Serious comorbidities requiring longer preoperative hospitalization can make it difficult to optimize patients’ conditions and preserve functional capacity before surgery. It has been recently demonstrated that a decline in preoperative functional capacity, determined by the Duke score activity index, is associated to increased myocardial infarction and death 30 days and one year after major non-cardiac surgery [49]. Prehabilitation, as preoperative optimization of patients’ condition, is a key ERAS concept [50]. However, it is evident that this should not prolong the preoperative hospital stay. Our analysis supports the hypothesis that preoperative hospital stay may even impair prehabilitation. Our results on the adverse effect of blood transfusion on outcome support the evidence that a careful management of preoperative anemia through iron supplemental therapy can improve overall morbidity and mortality by reducing the need for blood transfusion in the perioperative period [39, 51].

A possible limitation of our study is related to conceivable differences between the participating hospitals and through the time span of the data collection, both in the degree of ERAS pathway implementation and in the baseline standard of care. A potential selection bias could be at stake, despite all centers have been invited to submit consecutive elective patients. Nevertheless, the wide mix of ages and comorbidities included indicates a small likelihood of selection bias among patients.

The major strength of our proposal resides in the features of the proposed statistical method. Moreover, we accessed a dedicated ERAS database from which we derived the data used for the analyses and we used a validated indicator of short-term recovery such as the TRD [10].

## Conclusion

We analyze a colonic surgery ERAS database by proposing a multivariate causal latent class two-parameter logistic model. This modern statistical method overcomes several drawbacks of traditional methods to estimate average treatment effects on the treated. Since we are dealing with observational studies we employ a propensity score method. We propose to use a maximum likelihood approach by employing weights estimated through the inverse-probability of receiving the treatments. In this way, we avoid rough comparisons of patients thus producing a valid inference and reducing the possibility of biased treatment effects. As noted by [52] generally a simple covariate adjustment cannot be able to produce unbiased estimates of the model parameters. The proposed method of analysis is able to account for patient heterogeneity and it constitutes a general approach for the analysis of data arising in similar medical contexts.

According to the results early postoperative ambulation and IV fluid stop, epidural anesthesia, and waiving any surgical drainage exert a favorable effect on primary outcomes (time ready for discharge and actual discharge from hospital, and occurrence of any complication, surgical site infection, and medical complications), while prolonged preoperative hospital stay and blood transfusion act unfavorably.
